# Anatomical adjustments of the tree hydraulic pathway decrease canopy conductance under long-term elevated CO_2_

**DOI:** 10.1093/plphys/kiac482

**Published:** 2022-10-17

**Authors:** Marielle Gattmann, Scott A M McAdam, Benjamin Birami, Roman Link, Daniel Nadal-Sala, Bernhard Schuldt, Dan Yakir, Nadine K Ruehr

**Affiliations:** Institute of Meteorology and Climate Research – Atmospheric Environmental Research, Karlsruhe Institute of Technology, Garmisch-Partenkirchen 82467, Germany; Department of Botany and Plant Pathology, Purdue Center for Plant Biology, Purdue University, West Lafayette, Indiana 47907, USA; Institute of Meteorology and Climate Research – Atmospheric Environmental Research, Karlsruhe Institute of Technology, Garmisch-Partenkirchen 82467, Germany; Ecophysiology and Vegetation Ecology, Julius-von-Sachs-Institute of Biological Sciences, University of Würzburg, Würzburg 97082, Germany; Institute of Meteorology and Climate Research – Atmospheric Environmental Research, Karlsruhe Institute of Technology, Garmisch-Partenkirchen 82467, Germany; Ecophysiology and Vegetation Ecology, Julius-von-Sachs-Institute of Biological Sciences, University of Würzburg, Würzburg 97082, Germany; Department of Environmental Sciences and Energy Research, Weizmann Institute of Science, Rehovot 76100, Israel; Institute of Meteorology and Climate Research – Atmospheric Environmental Research, Karlsruhe Institute of Technology, Garmisch-Partenkirchen 82467, Germany; Institute of Geography and Geoecology, Karlsruhe Institute of Technology, Karlsruhe 76131, Germany

## Abstract

The cause of reduced leaf-level transpiration under elevated CO_2_ remains largely elusive. Here, we assessed stomatal, hydraulic, and morphological adjustments in a long-term experiment on Aleppo pine (*Pinus halepensis*) seedlings germinated and grown for 22–40 months under elevated (eCO_2_; c. 860 ppm) or ambient (aCO_2_; c. 410 ppm) CO_2_. We assessed if eCO_2_-triggered reductions in canopy conductance (*g*_c_) alter the response to soil or atmospheric drought and are reversible or lasting due to anatomical adjustments by exposing eCO_2_ seedlings to decreasing [CO_2_]. To quantify underlying mechanisms, we analyzed leaf abscisic acid (ABA) level, stomatal and leaf morphology, xylem structure, hydraulic efficiency, and hydraulic safety. Effects of eCO_2_ manifested in a strong reduction in leaf-level *g*_c_ (−55%) not caused by ABA and not reversible under low CO_2_ (c. 200 ppm). Stomatal development and size were unchanged, while stomatal density increased (+18%). An increased vein-to-epidermis distance (+65%) suggested a larger leaf resistance to water flow. This was supported by anatomical adjustments of branch xylem having smaller conduits (−8%) and lower conduit lumen fraction (−11%), which resulted in a lower specific conductivity (−19%) and leaf-specific conductivity (−34%). These adaptations to CO_2_ did not change stomatal sensitivity to soil or atmospheric drought, consistent with similar xylem safety thresholds. In summary, we found reductions of *g*_c_ under elevated CO_2_ to be reflected in anatomical adjustments and decreases in hydraulic conductivity. As these water savings were largely annulled by increases in leaf biomass, we do not expect alleviation of drought stress in a high CO_2_ atmosphere.

## Teaser

Increases in atmospheric CO_2_ can trigger structural modifications in leaves and branches, which contribute to lower leaf-level water loss, but do not affect the drought sensitivity in Aleppo pine.

## Introduction

Decreases in transpiration and stomatal conductance are among the most widely documented effects of elevated atmospheric [CO_2_] on plants (Drake et al., 1997; Medlyn et al., 2001; Ainsworth and Rogers, 2007; Domec et al., 2017; Dusenge et al., 2019; Birami et al., 2020; Poorter et al., 2022). Given that drought spells and extreme weather events are increasing with climate change (Seneviratne et al., 2021), quantifying that the extent to which increased CO_2_ reduces plant water loss has been the objective of numerous studies over the past two decades. Results indicate that elevated CO_2_ could potentially mitigate the negative effects of drought and heat in many plant species, although the extent varies depending on the type, severity, and duration of the stress (Huang and Xu, 2015; Brodribb et al., 2020). Contrastingly, it has also been observed that leaf-level responses—most prominently water savings from reduced stomatal conductance under elevated CO_2_—could be counterbalanced at the plant level due to enhanced leaf growth at higher CO_2_ (Tor‐ngern et al., 2015; Knauer et al., 2017; Jin et al., 2018; Gattmann et al., 2021). While the body of literature on plant responses to elevated CO_2_ is growing, major knowledge gaps persist (De Kauwe et al., 2021), particularly in terms of understanding the mechanisms driving the [CO_2_] effect on stomatal conductance (Poorter et al., 2022).

Addressing the processes that limit stomatal conductance under elevated CO_2_ is of utmost importance in a rapidly changing climate (Ainsworth and Rogers, 2007; Bonan, 2008; Jasechko et al., 2013; Klein et al., 2019). In angiosperms, there is a well-described instantaneous stomatal response to changes in atmospheric CO_2_ concentration (Morison, 1985, 1987; Mott et al., 2008). Stomata in most angiosperm species will open when exposed to [CO_2_] lower than ambient, and close when exposed to [CO_2_] higher than ambient. The mechanism driving these responses remains relatively elusive, although recent molecular work in Arabidopsis (*Arabidopsis thaliana*) suggests that a network of core and peripheral guard cell signaling pathways drive stomatal responses to elevated CO_2_ (Dubeaux et al., 2021). Nothing is known about the molecular signaling pathway for stomatal responses to [CO_2_] outside of angiosperms and there appears to be a considerable evolutionary transition in stomatal responsiveness to [CO_2_] across the land plant phylogeny, with angiosperm species generally having a much greater stomatal sensitivity to instantaneous changes in [CO_2_] in comparison to other stomata-bearing land plants (Doi and Shimazaki, 2008; Brodribb et al., 2009; Brodribb and McAdam, 2013; Haworth et al., 2013; Franks and Britton‐Harper, 2016; Kubásek et al., 2021). In angiosperms, the magnitude and speed of stomatal responses to an instantaneous change in [CO_2_] are regulated by abscisic acid (ABA) levels, with enhanced responses occurring in leaves with high ABA levels (Raschke, 1975; Dubbe et al., 1978; McAdam et al., 2011; Chater et al., 2015). This augmentation of stomatal sensitivity to instantaneous changes in [CO_2_] does not occur in conifers (McAdam et al., 2011), but it has never been examined whether ABA might play a role in regulating stomatal sensitivity to long-term increases in [CO_2_] in conifers.

The regulation of stomatal aperture and water loss is not restricted to physiological responses but could be altered by leaf anatomical adjustments after long-term exposure to high CO_2_. As widely observed, growth under elevated CO_2_ may result in reduced development of stomatal complexes in the epidermis, reducing both stomatal density (SD; number of stomata per unit leaf area) and stomatal index (SI; the proportion of epidermal cells that are stomata) (Woodward and Kelly, 1995). This anatomical adjustment reduces overall stomatal conductance and increases water-use efficiency (WUE) without a change in stomatal aperture. To date, results are far from conclusive, and often have no effect, and in some cases, even an increase in SD in response to elevated CO_2_ has been observed (Pritchard et al., 1999; Luomala et al., 2005; Domec et al., 2017). Hence, the magnitude of the SD response seems to be affected by the experimental setup and duration, species, and other environmental factors (Haworth et al., 2013; Xu et al., 2016). Contributing to lower SD under elevated CO_2_ could be a promotion of leaf size, as observed in grasses (Xu and Zhou, 2008; Xu et al., 2014), or an increase in needle thickness or width as observed in Scots pine (*Pinus sylvestris*) (Lin et al., 2001). These changes are often related to alterations in cell division and/or cell expansion driven mainly by increased carbon availability combined with a reduction in water demand (Pritchard et al., 1999; Xu et al., 2016).

In agreement, a growing number of studies suggest that anatomical adjustments from long-term exposure to elevated CO_2_ are not restricted to stomata but may affect leaf hydraulic conductance (*K*_leaf_; Domec et al., 2009; Phillips et al., 2011). Increases in needle thickness and/or mesophyll tissue (Lin et al., 2001), which affect the path length the water has to travel from the vein to the stomata, have been attributed to lower *K*_leaf_ (Domec et al., 2016), and could be related to lower stomatal conductance as well as an enhanced WUE (Trueba et al., 2022). Moreover, a few studies have observed adjustments in xylem structure in response to elevated CO_2_ (Domec et al., 2017). In conifers, structural changes were mixed and seem to vary among species, showing no responses (Maherali and DeLucia, 2000), increases in cell wall thickness (Conroy et al., 1990; Atwell et al., 2003; Kilpelainen et al., 2007; Domec et al., 2016), wood density (Telewski et al., 1999; Atwell et al., 2003), or tracheid diameter (Ceulemans et al., 2002). A recent literature review summarizes the impacts of some of these findings on tree hydraulics but no clear picture of conifers emerged. It appears that specific conductivity, that is, hydraulic conductivity normalized by xylem cross-sectional area (*K*_s_), might slightly increase while plant hydraulic conductance and leaf water potential remain largely unchanged under elevated CO_2_ (Domec et al., 2017). As the tree water transport system from roots to leaves is tightly coordinated (Meinzer and Grantz, 1990; Santiago et al., 2004; Bartlett et al., 2016), stomatal conductance could be indirectly affected by anatomical adjustments of xylem porosity, leaf thickness, or vein-to-stomata distance. The linking element here is the water status at the site of stomatal evaporation, which is influenced in part by the hydraulic conductivity of branches and leaves, and by stomatal aperture (Bartlett et al., 2016). Furthermore, changes in whole-plant water transport capacities become likely if growth under elevated CO_2_ alters xylem anatomy. These might ultimately affect the vulnerability of trees to hydraulic failure (Domec et al., 2010).

Trees growing in seasonally dry environments could be particularly affected by CO_2_-induced changes that affect water demand and transport. In two previous studies, we have investigated the effect of elevated CO_2_ to heat, hot drought, and lethal drought in Aleppo pine (*Pinus halepensis*) seedlings (Birami et al., 2020; Gattmann et al., 2021) grown from seeds either under ambient (aCO_2_ c. 410 ppm) or highly elevated [CO_2_] (eCO_2_ c. 870 ppm) for 18–22 months. Seeds originated from the Yatir forest, a Aleppo pine dominated forest plantation at the northern edge of the Negev desert in Israel (Grünzweig et al., 2003). The experimental CO_2_ concentrations were within the range of the RCP8.5 scenarios (794–1,142 ppm for 2,100) and hence close to CO_2_ saturation for photosynthesis, providing a strong CO_2_ response. Results of these two previous studies showed that elevated CO_2_ enhanced whole-tree C uptake (c. +100%), WUE, and overall tree biomass (+35%). Pronounced reductions in leaf-level canopy conductance (*g*_c_) largely counterbalanced the increase in leaf area resulting in comparatively small water savings at the tree level (c. −10% at 25°C). Exposing the seedlings to heat or hot drought spells revealed little effect of elevated CO_2_ on the stress response, albeit maintaining a higher WUE until respiration rates exceed photosynthesis (Birami et al., 2020). Further, elevated CO_2_ did not improve the overall tree vulnerability to a lethal soil drought and the decline in leaf water potential, as well as thresholds for stomatal closure and turgor loss point, appeared unaffected (Gattmann et al., 2021). This raises the question of the underlying mechanisms that reduced *g*_c_ under elevated CO_2_ and why the physiological drought response remained largely unaltered.

**Figure 1:**
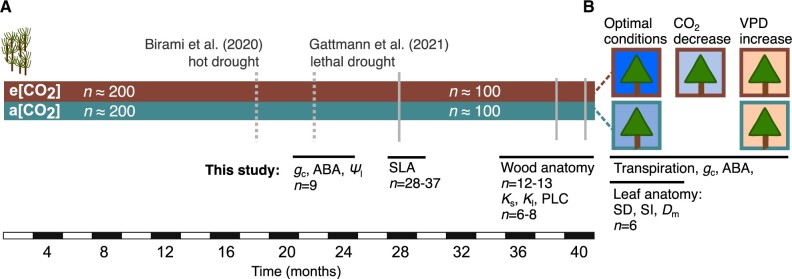
Timeline of cultivation of *P. halepensis* seedlings under ambient (c. 400 ppm) or (c. 860 ppm) elevated CO_2_. A, The number of seedlings cultivated after germination, the conducted experiments and references, as well as measurements related to this study are shown. B, After 40 months cultivation, randomly selected seedlings (*n* = 6 per treatment) were placed into automated gas exchange chambers enclosing the canopy of one seedling each and transpiration and *g*_c_ were measured during optimal conditions, decreasing CO_2_ (in eCO_2_ seedlings only) and increasing VPD over several days alongside which leaf samples were taken to analyze for ABA content. B, This chamber system was also used to measure responses in the same population of seedlings during a dry-down experiment on 22-month-old seedlings when also leaf water potential (Ψ_l_) was measured as published in Gattmann et al. (2021). Changes in leaf morphology were assessed via analyses of specific leaf area (SLA), SD, SI, and vein-to-epidermis distance (*D*_m_). Measurements of wood anatomy included conduit diameter, lumen fraction, and potential hydraulic conductivity. Changes in hydraulic properties were assessed via measurements of specific conductivity (*K*_s_), leaf-specific conductivity (*K*_l_) and PLC.

Here, we take advantage of this long-term elevated CO_2_ experiment and assessed the coordination between anatomical and physiological adjustments of Aleppo pine seedlings from the same population grown for 22–40 months under elevated CO_2_ averaging c. 860 ppm over the entire period. We studied leaf-level *g*_c_ responses to [CO_2_] and increasing soil or atmospheric drought and assessed if those are coordinated by morphological changes in the hydraulic system (Figure 1). We addressed the following hypotheses: (1) stomatal closure under eCO_2_ is reversible upon exposure to low CO_2_ if a direct stomatal response, possibly mediated by the phytohormone ABA; (2) stomatal closure under eCO_2_ is not reversible as hydraulic conductance is modified by anatomical adjustments of leaves and wood, specifically reductions in xylem tracheid diameter and SD; and (3) stomatal responses to drought and increasing vapor pressure deficit (VPD) are unaffected by elevated CO_2_ and hence hydraulic vulnerability is unchanged.

## Results

### Long-term acclimation to elevated CO_2_ affects gas exchange

**Figure 2:**
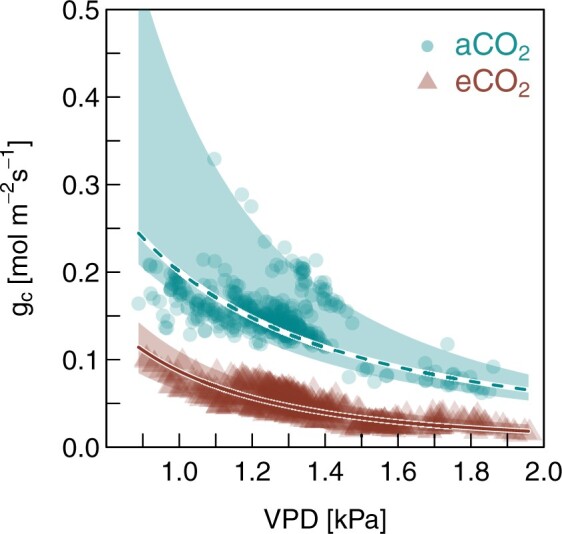
Treatment-specific relationships of *g*_c_ with VPD for 40-month-old *P. halepensis* seedlings grown under ambient or elevated CO_2_. Shown are single measurements for six trees per treatment (aCO_2_: circles; eCO_2_: triangles) over the course of 3 days for PAR > 200 µmol m^−2^ s^−1^. The trend line gives the median value of the model fit and the shaded areas represent the 95% CIs per treatment (see Supplemental Table S2 for model coefficients).

**Figure 3:**
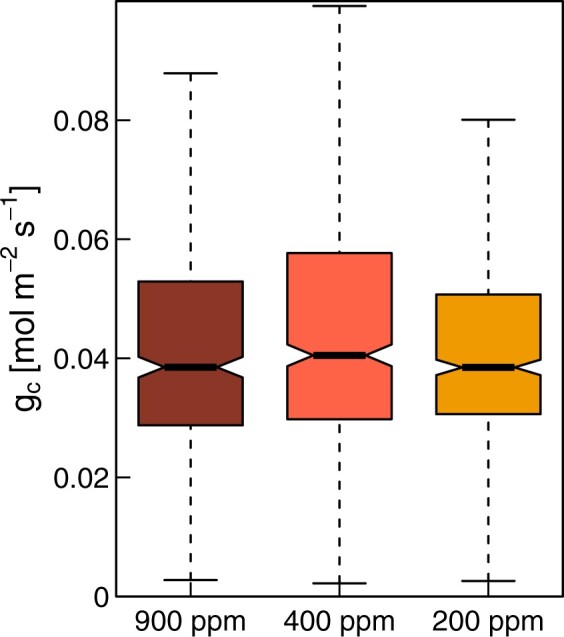
*g*_c_ measured at three different CO_2_ concentrations of Aleppo pine seedlings grown for 40 months under elevated CO_2_ (*c*. 870 ppm). In order to test whether reduced [CO_2_] can trigger stomatal opening, the CO_2_ seedlings (*n* = 6) were steadily exposed to decreasing atmospheric [CO_2_] from *c*. 900, to *c*. 400, and *c*. 200 ppm and each CO_2_ level was kept for 2 days. Shown are boxplots of the automated gas exchange measurements for six seedlings at PAR > 200 µmol m^−2^s^−1^. The box represents the interquartile range (IQR) of the data, the horizontal line at the notch is the median and the whiskers are 1.5 times the IQR. The number of measurements included were *n* = 481, *n* = 584, and *n* = 592 at 900, 400, and 200 ppm, respectively. Differences between CO_2_ levels tested with linear mixed effect models were not significant (*P* > 0.05).

Highly elevated atmospheric [CO_2_] strongly reduced leaf-level gas exchange rates in Aleppo pine seedlings compared to seedlings under ambient [CO_2_] (Supplemental Figure S1, a and b). These reductions were noticeable both during daytime and nighttime and *g*_c-ref_ (*g*_c_ at a VPD of 1 kPa) was 55% lower in eCO_2_ compared to aCO_2_ seedlings (Table 1). But the dynamics of the diurnal cycle were not altered by the CO_2_ treatment, suggesting that on a relative scale, *E* and *g*_c_ were affected similarly by short-term changes. This assumption was reinforced by the lack of a treatment effect in the slope of *g*_c_ responding to increasing VPD (Figure 2). In addition, reducing atmospheric [CO_2_] from 900 to 400 and 200 ppm did not result in higher *g*_c_ in eCO_2_ seedlings (Figure 3).

**Table 1:** Treatment responses of leaf morphology, stomatal characteristics, reference stomatal conductance at VPD = 1 kPa (*g*_c-ref_), and hydraulic vulnerability parameters for *P. halepensis* seedlings grown for 40 months under aCO_2_ or eCO_2_ atmospheric CO_2_

Trait, unit	aCO_2_	eCO_2_
SL, **μ**m	51.87 ± 5.89^(a)^	55.70 ± 8.53^(a)^
**SD, n mm** ^−2^	28.92 ± 3.81^(a)^	35.46 ± 4.75^(b)^
**ED, n mm** ^−2^	133.20 ± 15.20^(a)^	166.67 ± 20.13^(b)^
SI, %	17.87 ± 1.71^(a)^	17.51 ± 1.29^(a)^
***D*_m_, µm**	150.97 ± 9.65^(a)^	252.82 ± 6.67^(b)^
**LW, mm**	0.85 ± 0.05^(a)^	1.19 ± 0.05^(b)^
ABA, ng g^−1^ FW	305 ± 93^(a)^	169 ± 149^(a)^
**LA, cm^2^**	0.74 ± 0.21^(a)^^$^	0.87 ± 0.22^(b),#^
**SLA, cm^2^ g^−1^**	55.48 ± 6.01^(a)^^$^	51.04 ± 8.84^(b),#^
***g*_c-ref_, mol m^−2^ s^−1^**	0.2 [0.17–0.41]^(a)^	0.09 [0.07–0.11]^(b)^
Ψ_gc-close_, −MPa	2.1 [2.20–2.00]^(a)^	2.15 [2.25–2.00]^(a)^
*P*_12_, −MPa	3.92 [4.24–2.92]^(a)^	3.33 [4.13–2.35]^(a)^
*P*_50_, −MPa	5.17 [5.88–4.66]^(a)^	4.91 [5.96–4.19]^(a)^
*P*_88_, −MPa	6.11 [7.79–5.97]^(a)^	6.66 [7.79–5.37]^(a)^

Stomata length (SL), SD, number of ED, SI LW, and vein-to-epidermis distance (*D_m_*), ABA, needle leaf area (LA) and SLA are mean ± sd (*n* = 6 per treatment if not noted otherwise). Statistical significance was tested with nonparametric Mann–Whitney U tests (*P* < 0.05). For *g*_c-ref_, Ψ_leaf_ at stomatal closure (Ψ_gc-close_) and the hydraulic vulnerability parameters (*P*_12_, *P*_50_, and *P*_88_), the treatment median with the 95% CIs of the Bayesian model fit is given. Parameters with significant differences between treatments are highlighted in bold and indicated by different letters.

$ aCO_2_*n* = 28, ^#^ eCO_2_*n* = 37 measured in 28-month-old seedlings.

### Stomata control and characteristics under elevated CO_2_

**Figure 4:**
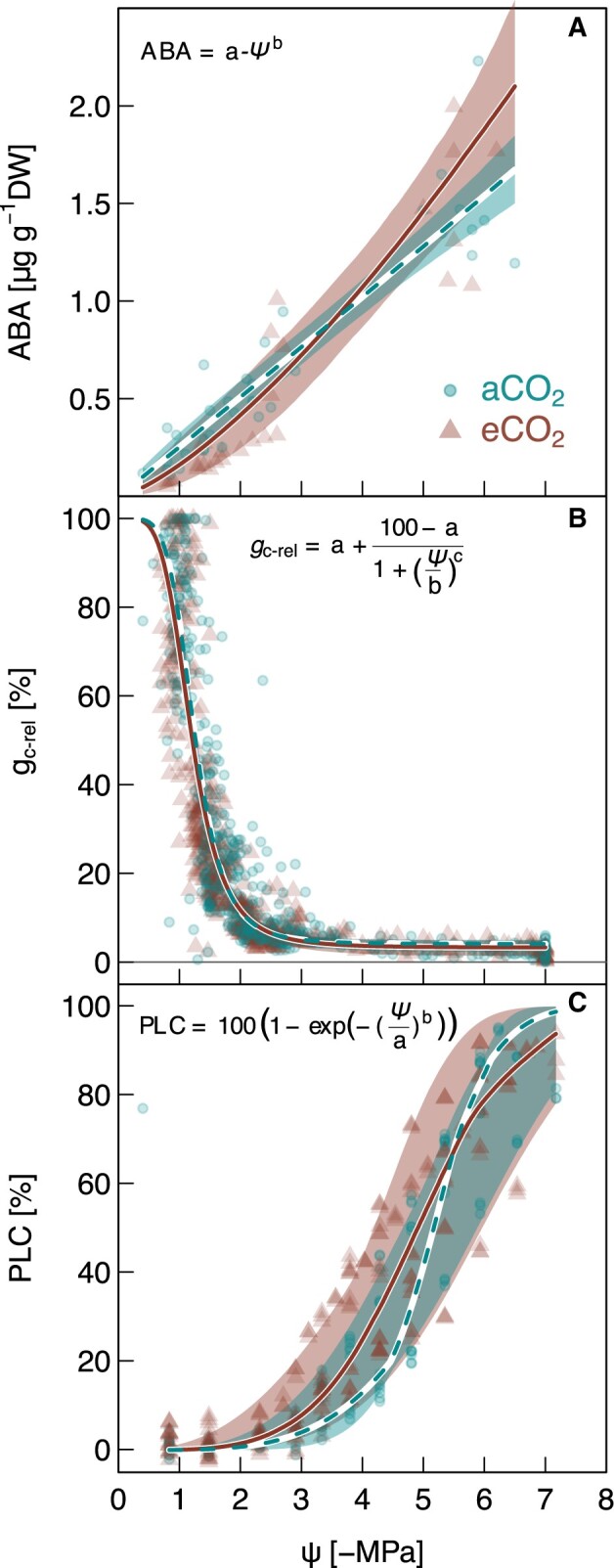
Hydraulic responses to increasing drought in *P. halepensis* seedlings grown under ambient or elevated CO_2_. Treatment-specific relationships of (A) leaf-level ABA concentration given per dry weight (DW) needle tissue and (B) relative *g*_c_ (*g*_c-rel_) with leaf water potential (Ψ) during a dry-down experiment are shown for the 22-month-old seedlings (*n* = 6 per treatment), and (C) PLC in branches versus xylem water potential (Ψ) is given for 40-month-old seedlings (aCO_2_*n* = 6 eCO_2_*n* = 8). Data points of individual measurements (aCO_2_: solid points, eCO_2_: solid triangles) are shown. The median of each model is indicated by solid (eCO_2_) or dashed (aCO_2_) lines and the shaded areas represent the 95% credible intervals per treatment (see Supplemental Table S2 for model coefficients). For values of *P*_12_, *P*_50_, and *P*_88,_ see Table 1.

Elevated CO_2_-induced reductions in *g*_c_ were not driven by higher leaf ABA levels (Table 1). Moreover, the lack of a CO_2_ effect on ABA levels was further confirmed as ABA levels increased with decreasing Ψ_leaf_ in both eCO_2_ and aCO_2_ seedlings at the same rate (Figure 4A). In addition, the stomatal aperture was tightly regulated by leaf water status, independent of the CO_2_ treatment, as shown by the similar steep decline of relative *g*_c_ with Ψ_leaf_ in both treatments (Figure 4B, see also Supplemental Figure S3 for Ψ_leaf_ during dry-down). The decline in *g*_c_ with decreasing Ψ_leaf_ and the water potential at stomatal closure was comparable in both treatments (c. −2.1 MPa; Figure 4, B and C and Table 1).

To assess possible morphological adjustments in response to elevated CO_2_ that could account for the reduced *g*_c_ in eCO_2_ plants, we analyzed leaf and stomata morphology (Table 1). We found that growth under elevated CO_2_ resulted in a significant increase in SD and epidermal pavement cell density (ED) (Table 1). As these increases in SD (+23%) and ED (+25%) were similar, there was no significant change in SI induced by elevated CO_2_. We also analyzed needle width and vein-to-epidermis distance and found both to be significantly larger in leaves adapted to elevated CO_2_ (Table 1). These increases in needle width corresponded to increases in individual leaf area (Table 1).

### [CO_2_] effect on wood anatomy and hydraulic parameters

**Figure 5:**
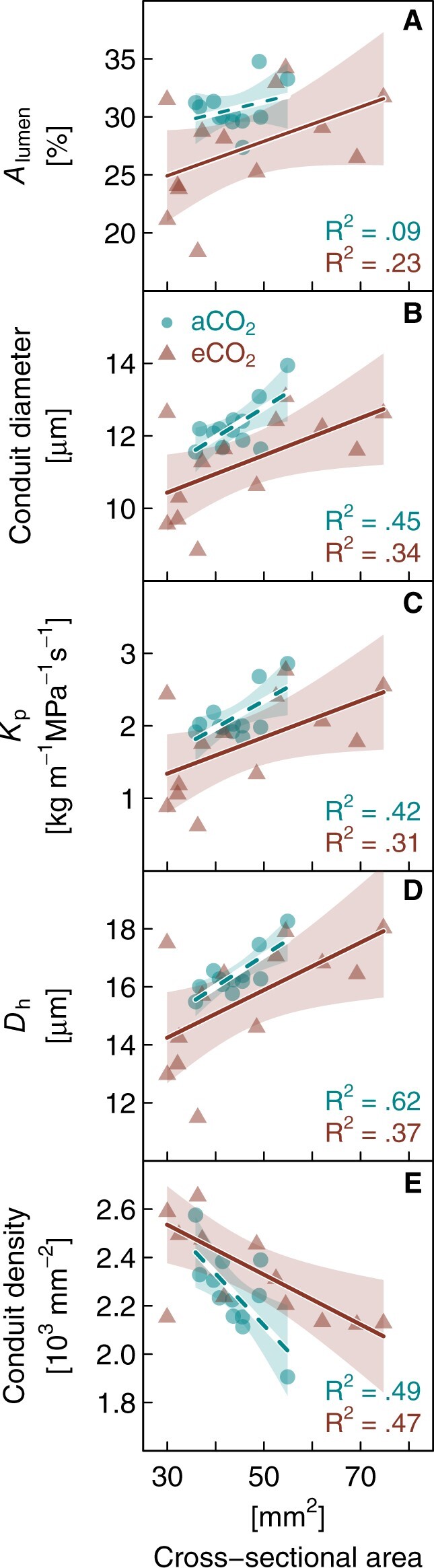
Wood anatomy parameters in relation to branch cross-sectional area in *P. halepensis* seedlings grown for 40 months under eCO_2_ or aCO_2_. A, Lumen fraction (*A*_lumen_), (B) average conduit diameter, (C) potential conductivity (*K*_p_), (D) hydraulically weighted conduit diameter (*D*_h_), and (E) CD are given. Data are measurements of individual branch samples (*n* = 12–13 per treatment). Linear regressions (aCO_2_: intermitted lines, eCO_2_: solid lines) and the 95% confidence intervals of the fit are given (shaded areas). Note that differences between treatments were significant for *A*_lumen_, conduit diameter and *K*_p_ (generalized least squares test, *P* < 0.05).

Elevated CO_2_ altered the woody anatomy of branches. As cross-sectional area had a substantial influence on all wood anatomical parameters and the samples from the different treatments differed systematically in average diameter, it was included as a covariate in the evaluation of the CO_2_ effects (Figure 5). Comparing hydraulic parameters between CO_2_ treatments including cross-sectional area revealed significant reductions in conduit lumen fraction (*A*_lumen_, −11%; Figure 5A), average conduit diameter (*D*, −8%; Figure 5B), and potential conductivity (*K*_p_, −17%; Figure 5C) of eCO_2_ seedlings (generalized least square [GLS], *P* < 0.05). Conduit density (CD; Figure 5D) tended to be on average ∼5% (*P* = 0.057) higher in eCO_2_ than aCO_2,_ while the hydraulically weighted conduit diameter (*D*_h_, Figure 5D) was not affected by growth CO_2_.

**Figure 6:**
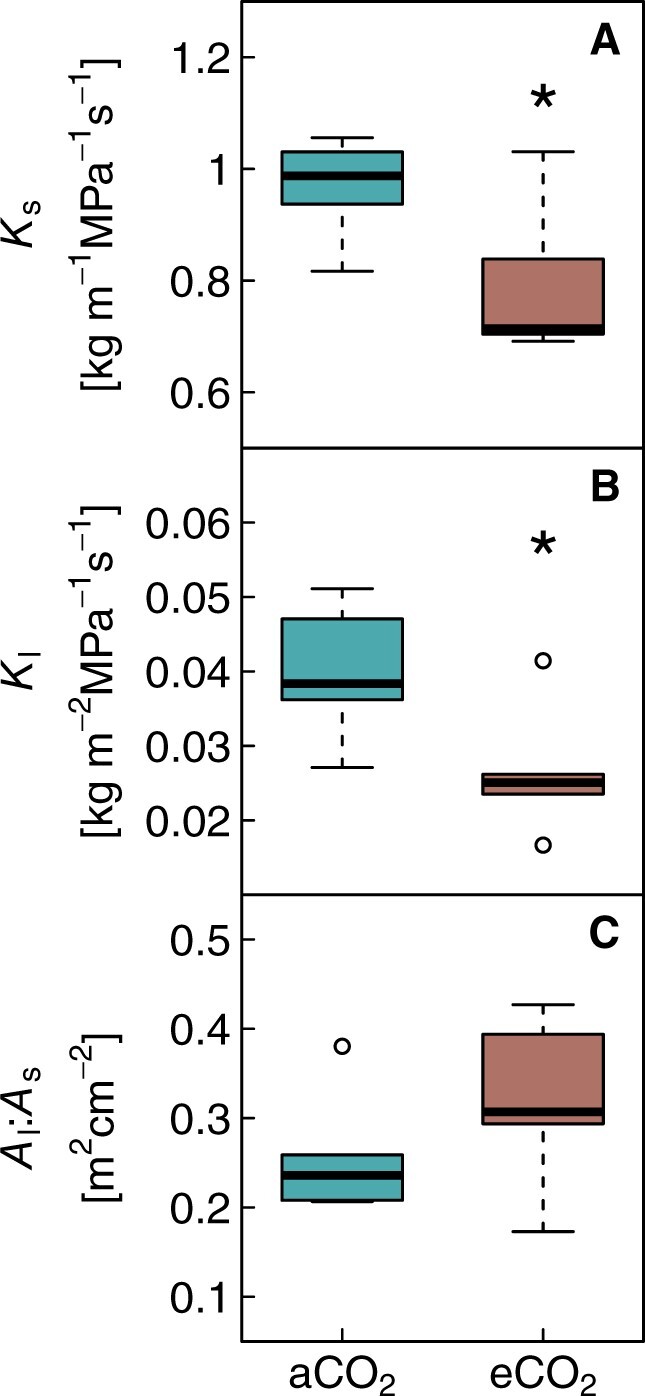
Responses of hydraulic conductivity and leaf-to-sapwood area in 40-month-old *P. halepensis* seedlings grown under ambient or elevated CO_2_. A, Specific conductivity (*K*_s_), (B) leaf specific conductivity (*K*_l_), and (C) leaf-to-sapwood area ratio (*A*_l_:*A*_s_) are shown as boxplots per treatment (*n* = 6). The box represents the IQR of the data, the horizontal line inside the box is the median, the whiskers cover 1.5 times the IQR. Data points are outliers beyond the extremes of the whiskers. Significant treatment differences are indicated by asterisks (Mann–Whitney U test *P* < 0.05).

The distinct signal of morphological changes under elevated CO_2_ was also captured in specific (*K*_s_) and leaf-specific (*K*_l_) conductivity. Both were significantly reduced in eCO_2_ plants (*K*_s_: −19%; *K*_l_: −34%; Mann–Whitney U test, *P* < 0.05; Figure 6, A and B). The leaf-to-sapwood area ratio (*A*_l_:*A*_s_, Figure 6C) tended to be larger (+24%, *P* = 0.090) in eCO_2_ compared to aCO_2_ seedlings.

## Discussion

### Stomatal responses to elevated CO_2_

Aleppo pine seedlings grown from seeds for 40 months under highly elevated CO_2_ had a 55% lower leaf-level *g_c_* than aCO_2_ seedlings (Table 1). This reduction was 15% larger when compared to results from our previous study (Birami et al., 2020), possibly affected by the duration of CO_2_ fumigation or seasonal timing of the experiment. Overall, the observed reductions in *g_c_* exceed the average decrease of about 20% reported in many other tree species grown under elevated CO_2_ (Medlyn et al., 2001; Ainsworth and Rogers, 2007; Purcell et al., 2018). These differences could be due to the high CO_2_ levels of 860 ppm in our study, compared to the average of c. 570 ppm in previous studies (Ainsworth and Rogers, 2007). To further investigate the mechanism driving this considerable reduction in leaf-level *g_c_* in Aleppo pine seedlings, we first conducted an experiment to test whether the reduced *g_c_* was driven by a direct stomata response (Ainsworth and Rogers, 2007; Xu et al., 2016; Gamage et al., 2018; Birami et al., 2020). We found that stomata in elevated CO_2_-adapted plants did not respond to [CO_2_] reductions from c. 900 to 400 and 200 ppm with each level kept for 2 days (Figure 3). This suggests that stomata were not actively closed by the high CO_2_ level; rather, the stomata were likely operating at an optimal level that was physiologically and anatomically determined. A similar result has been observed after 17 years of CO_2_ enrichment (+200 ppm) at the DUKE FACE site, in which [CO_2_] was decreased step wise, without any indications of *g*_c_ to respond in a Loblolly pine (*Pinus taeda*) dominated stand (Tor‐ngern et al., 2015). In our study, we further showed that ABA levels were not causing a direct decline of *g*_c_ under elevated CO_2_, or augmenting this response as observed in angiosperms (Dubbe et al., 1978). This reinforces the assumption that the *g*_c_ reduction might be indirect (via developmental changes) rather than direct (via active stomatal aperture adjustment) and hence rejects our first hypothesis. While this contrasts observations in most angiosperm species (Giday et al., 2014; Chater et al., 2015), it is in line with previous studies which have found that conifers lack considerable instantaneous stomatal closing responses to elevated CO_2_ (Brodribb et al., 2009; Brodribb and McAdam, 2013; Haworth et al., 2013).

As our 40-month-old Aleppo pine seedlings had grown their entire life under elevated CO_2_, indirect responses via anatomical adjustments might be the most prominent explanation on apparent declines in *g*_c_. We found epidermal developmental adjustments to elevated CO_2_, particularly an increased SD and ED (Table 1). This increase in SD at elevated CO_2_ has rarely been documented (Ferris, 1996; Reid, 2003; Zhou et al., 2013) and is contrary to our expectation of SD reductions (Lin et al., 2001; Kouwenberg et al., 2003; Haworth et al., 2011) or no changes in SD (Apple et al., 2000; Kurepin et al., 2018). While SD and ED increased, we found SI to remain unaffected (Table 1), rejecting our hypothesis that—as in many angiosperms—a reduced initiation of stomata occurred in response to elevated CO_2_ in Aleppo pine seedlings. As SD did not change independently of epidermal cell density, we suggest that the observed increase in SD was mediated by a decrease in epidermal cell expansion that led to a smaller final size of epidermal cells. Decreased epidermal cell size has been reported previously in *Phaseolus vulgaris* exposed to elevated CO_2_ due to changes in both cell division and expansion (Ranasinghe and Taylor, 1996). So far, the underlying mechanisms of increased leaf cell production under elevated CO_2_ are not fully resolved, but could potentially be triggered by a surplus of carbohydrates and may be linked to the increase in individual leaf size we observed in eCO_2_-adapted plants.

### Hydraulic conductance in leaves and branches under elevated CO_2_

We found needles of eCO_2_ seedlings to increase in width and cross-sectional area consequently having a longer distance for water to traverse from the vein to the substomatal cavity. Increasing the radial path length for water transport effectively limits *K*_leaf_ (Brodribb et al., 2010). A larger distance from the vein-to-the-epidermis has been shown to be linearly related to *K*_leaf_ in a range of species (Brodribb et al., 2007). This supports that *K*_leaf_, although not measured directly in our study, must have been lower in eCO_2_ seedlings. Moreover, an increased vein-to-epidermis distance has also been reported after long-term exposure to elevated CO_2_ in Loblolly pine, which was directly related to reduced *K*_leaf_ and further attributed to increased resistance of outside xylem water transport (Domec et al., 2016). Similar to our study, stomata did not open when CO_2_ was reduced (Tor‐ngern et al., 2015). In addition, elevated CO_2_ increased the needle cross-sectional area and mesophyll surface area in Scots pine (Lin et al. 2001), and recently it has been shown that a lower stomata-to-mesophyll volume ratio relates to lower stomatal conductance in conifers (Trueba et al., 2022). In Aleppo pine we found the increase in vein-to-epidermis distance (+65%) to be proportionally larger than the increase in SD (+23%), eventually contributing to a reduced *g*_c_ under elevated CO_2_. Conifer leaves are not fully vascularized and as a consequence exhibit a larger difference in water potentials between veins, mesophyll, and epidermis, which may result in stomata closure even when xylem water potential is relatively high (Zwieniecki et al., 2007). Albeit leaf water potential did not differ between CO_2_ treatments (Supplemental Figure S2), undetected, minor changes in localized water potential—eventually restricted to stomatal guard cells—could have resulted in turgor-driven partial stomatal closure in these conifers (McAdam and Brodribb, 2014). Additional work examining the impact of potentially altered mesophyll anatomy, transfusion tissue area, and cell size on *K*_leaf_ and stomatal conductance is warranted. In addition, undetected morphological changes of the stomata structure, including a greater stomatal pore depth or increases in cuticular waxes, may contribute to reduced transpiration.

We found branches of the eCO_2_ seedlings to have a lower specific conductivity (*K*_s_) and reduced leaf-specific conductivity (*K*_l_). This was manifested in xylem morphology, reflected in a lower conduit lumen fraction and reduced average conduit diameter in branches under elevated CO_2_. These anatomical changes to leaf morphology and xylem structure have likely manifested during early seedling development triggered by reduced stomatal aperture and lower water demand under high CO_2_. We did not find a clear signal just a tendency of increasing leaf-to-sapwood area under eCO_2_ in line with a study on cottonwood trees grown under highly elevated CO_2_ (1,200 ppm) (Engel et al., 2004). This suggests that trees under elevated CO_2_ allocate comparably less resources into tree water transport but more into leaf structure. At the tree level, this resulted in a larger leaf area and increased C uptake in eCO_2_ plants (Supplemental Figure S3b).

These anatomical responses, as depicted, should need relatively long exposure times to elevated CO_2_. For instance, in experiments on mature trees, such responses might not become apparent, as CO_2_ fumigation typically spans few growing seasons and most of the woody tissue has been formed previously (e.g. Körner, 2006). This indicates that particularly in diffuse-porous and conifer species, which conduct water through multiple tree rings (Maton and Gartner, 2005), xylem hydraulic responses might only manifest after a large fraction of the woody tissue has been grown under elevated CO_2_ conditions, and hence might have not been routinely observed in previous studies (Domec et al., 2017). In contrast, responses at the leaf-level should develop more quickly (e.g.Tor‐ngern et al., 2015). In our study we found, albeit anatomical adjustments in leaves and xylem strongly reducing leaf-level water loss, no differences in Ψ_leaf_ and a minor response of tree-level transpiration (Supplemental Figures S2 and S3a). The reason is a pronounced increase of leaf area under eCO_2_ that largely annulled leaf-level water savings (Gattmann et al., 2021). In summary, this indicates a tight coordination between anatomical adjustments and water demand in Aleppo pine seedlings grown their entire life time in a highly enriched CO_2_ atmosphere.

### Implications for drought and VPD responses under elevated CO_2_

The rate of stomatal closure during increasing soil or atmospheric drought was not affected by elevated CO_2_, albeit *g*_c-ref_ (*g*_c_ at VPD of 1 kPa) being 55% lower in eCO_2_ plants. Increasing VPD from 1 to 2 kPa resulted in a 60% reduction of *g*_c_ in both treatments. This similar *g*_c_ behaviour was reflected in ABA levels increasing as Ψ_leaf_ declined (Figure 4), and supports previous findings of an unchanged physiological drought response in Aleppo pine grown under elevated CO_2_ (Birami et al., 2020; Gattmann et al., 2021). In agreement, the hydraulic vulnerabilities reported here, indicate no differences in *P*_12_, *P*_50_, or *P*_88_ values between treatments or the water potential at stomatal closure, which supports our last hypothesis. However, it is worth mentioning that the uncertainties were relatively large and a higher number of samples would have provided larger confidence in these results. Albeit hydraulic safety was not affected, we found hydraulic efficiency to decrease under eCO_2_, this was not surprising as hydraulic efficiency and safety are typically weakly linked (Gleason et al., 2016). A lower hydraulic conductivity was reflected in smaller conduits (−8%) and lower lumen fraction (−11%) of the xylem. Decreases in xylem porosity as we found under elevated CO_2_ are contradictory to findings in a few conifers, which apparently tend toward larger conduit diameter and less drought-resistant xylem (Domec et al., 2017). But the results on CO_2_ impacts on tree hydraulics and wood anatomy in conifers are generally mixed and sparse (Telewski et al., 1999; Olszyk et al., 2005; Domec et al., 2010) and no general picture emerges. Hence, it is worth noting that the CO_2_ effects on wood anatomy as found in our study were moderate and became apparent only after accounting for the confounding effect of branch cross-sectional area, which might not routinely be considered in other studies. In summary, while some of the observed morphological adjustments could be interpreted as a protective measure, neither the hydraulic vulnerability curves nor the *g*_c_ response to increasing soil or atmospheric drought indicates an increased hydraulic safety of Aleppo pine seedlings grown under elevated CO_2_.

Our study provides evidence of an unchanged metabolic and hydraulic stress response in pine seedlings grown under highly elevated CO_2_. The water savings from reduced transpiration were largely compensated by an increased leaf area so that tree-level water loss was marginally lower in the eCO_2_ treatment (Supplemental Figure S3) and the drought dynamics appeared unchanged (Gattmann et al., 2021). Based on these results, we suggest that drought responses of mature trees in the field should depend on leaf area stimulations from elevated CO_2_ (De Kauwe et al., 2021), which in turn affects tree and forest water demand. For instance, if an increase in leaf area balances CO_2_-induced reductions in water loss, tree or ecosystem-level drought progression should be unchanged. In contrast, if the leaf area does not respond to elevated CO_2_, the CO_2_-induced leaf-level water savings as observed here have the potential to buffer forest drought progression as soil water resources should deplete more slowly.

## Materials and methods

### Plant material

Aleppo pine seedlings were cultivated from seed either under ambient (on average 410 ± 23 ppm) or elevated (on average 860 ± 15 ppm) [CO_2_] for 40 months in a greenhouse facility in Garmisch-Partenkirchen, Germany (732 m a.s.l., 47°28′49.2″N, 35°3′7.2″E). Other environmental drivers such as air temperature (daytime c. 22°C ± 2.5°C, nighttime 15°C ± 2°C), relative humidity (75% ± 15%), photosynthetically active radiation (PAR; 480 ± 180 μmol m^−2^ s^−1^), and water availability (watered daily to full saturation) were maintained at similar levels in both CO_2_ treatments, and the mean difference between daily mean air temperature was typically <1°C. Winter periods (months December to February) were mimicked in the greenhouse by reducing daily air temperature to 12°C on average. The placement of the seedlings was repeatedly changed between and within the greenhouse compartments. During the initial 24 months of cultivation, the seedlings were placed in pots and repotted twice (last into 4.5-L pots). The potting substrate, a mixture of quartz sand, vermiculite, and expanded clay, was repeatedly enriched with slow-release fertilizer (Osmocote Exact Standard 5-6M 15-9-12 + 2MgO+TE; ICL Specialty Fertilizers) and supplemented by liquid fertilizer during the growing period. Initially, about 200 seedlings were grown under aCO_2_ or eCO_2_ to ensure a large enough population from which to randomly select seedlings for stress experiments involving destructive sampling. More details on seedling cultivation and experiments can be found in the two previously published studies, which used seedlings from the same population (Birami et al., 2020; Gattmann et al., 2021).

### Experimental setup and growth conditions

We assessed stomatal responses in eCO_2_- and aCO_2_-grown seedlings to drought, VPD, and decreasing [CO_2_] using custom-made gas exchange chambers (Birami et al., 2020; Gattmann et al., 2021; Rehschuh et al., 2022) consisting of a tightly sealed shoot compartment separated from the root compartment. In brief, each of the shoot compartments was made of a light-transmitting cylinder and was individually temperature controlled and equipped with temperature sensors (5SC-TTTI-36-2 M, Newport Electronics GmbH, Deckenpfronn, Germany). The drought experiment was conducted in early 2018 when the seedlings (*n* = 9 per treatment) were ∼22-month old (see Figure 6) as described in detail by Gattmann et al. (2021). The VPD and decreasing CO_2_ experiments were conducted later on 40-month-old seedlings in September 2019. Prior to experiments, we randomly selected six seedlings per treatment (each ∼50 cm tall). We conducted the experiments in a sequence starting with the eCO_2_ seedlings followed by aCO_2_ seedlings. Each seedling was placed into one of the six individual gas exchange chambers with the shoot compartment tightly sealed from the belowground part of the plant. Ambient sunlight was supplemented by plant growth lamps (T-agro 400 W; Philips, Hamburg, Germany), ensuring a relatively constant average PAR (PQS 1, Kipp & Zonen, Delft, the Netherlands) of 450 ± 50 μmol m^−2^ s^−1^ over each 16-h day measurement period (Supplemental Figure S1, c and d). All seedlings were automatically drip irrigated daily to field capacity.

### Gas exchange measurements

To test for reversibility in the restriction on *g*_c_ in eCO_2_ plants, [CO_2_] was reduced as follows. After the shoot of each seedling was placed into an individual gas exchange chamber, elevated CO_2_ was maintained for 3 days at 854 ± 29 ppm, then [CO_2_] was reduced close to ambient concentrations (382 ± 19 ppm) and then to 199 ± 9 ppm, maintaining each [CO_2_] change for 2 days. All other chamber conditions were kept constant with the day-time VPD at 1.34 ± 0.22 kPa and day-time air temperature at 24.2°C ± 0.49°C (min/max: 21.9°C/25.9°C). Nighttime temperature was maintained at 19.70°C ± 0.58°C. Temperature variations between chambers were small (<2°C).

We evaluated the responses of *g*_c_ in eCO_2_ seedlings to changes in VPD during 3 days under elevated CO_2_ (841 ± 23 ppm). VPD was allowed to vary diurnally from 0.9 kPa to 2.1 kPa while air temperature was maintained almost constant (min/max: 23.0°C/24.7°C). Following the removal of the eCO_2_ seedlings from the chambers, the aCO_2_ seedlings were installed, and *g*_c_ responses to VPD were assessed over three consecutive days at ambient CO_2_ (432 ± 17 ppm) with VPD ranging from 0.8 to 2.0 kPa, while air temperature was maintained relatively constant (min/max: 22.0°C/25.8°C).

Canopy H_2_O gas exchange (*n* = 6 per treatment) was derived by directly measuring absolute [CO_2_] and [H_2_O] of the 10 L min^−1^ supply air stream (LI-840, Li-cor, Lincoln, NE) and the concentration differences between supply and sample air stream (Li-7000, Li-cor, Lincoln, NE). Data were recorded every 10 s while the system automatically switched between chambers every 120 s so that each chamber was measured at least half-hourly. The last 40 s of each measurement was used to calculate net photosynthesis, transpiration, and *g*_c_. Two empty chambers were additionally integrated into the measurement cycle to continuously monitor the system and correct the data for any fluctuations in [H_2_O] that were not due to plant activity and these were typically small (0.03 ± 0.20 ppt H_2_O).

To quantify *g*_c_ to water vapor we derived leaf-level transpiration rate (*E*) in [mol m^−2^s^−1^] as follows:

(1) $$E=\frac{F_{m}(W_{\mathrm{supply}}-W_{\mathrm{sample}})}{A_{\mathrm{leaf}}(1-W_{\mathrm{sample}})},$$

where *W*_supply_ [mol mol^−1^] is [H_2_O] in supply air stream, *W*_sample_ [mol mol^−1^] is [H_2_O] in sample air stream, *F*_m_ [mol s^−1^] is molar flow and *A*_leaf_ [m^2^] is the two-dimensional leaf surface area of the shoot.

Canopy stomatal conductance (*g*_c;_ [mol H_2_O m^−2^ s^−1^]) was then calculated from leaf-level transpiration and water vapor concentration using the following equation:

(2) $$g_{c}=\frac{E\left(1-\frac{W_{\mathrm{leaf}}+W_{\mathrm{sample}}}{2}\right)}{W_{\mathrm{leaf}}-W_{\mathrm{sample}}},$$

where *W*_leaf_ is leaf saturated vapor pressure, derived from saturation vapor pressure (kPa) at a given air temperature (°C) and atmospheric pressure. Boundary layer conductance was neglected due to high mixing conditions generated from fans and high flow rates inside the chambers (Birami et al., 2020).

To determine leaf area at the end of the gas exchange measurements, all leaves were harvested, dried at 60°C for 48 h and weighed. Leaf biomass was then multiplied by specific leaf area, previously determined from a subsample of needles.

### Tissue sampling for anatomy and hormone analysis

Leaves for ABA quantification and epidermal anatomy were sampled randomly from each of the seedlings in the gas exchange chambers (*n* = 6 per treatment). The sampling was conducted between 12:00 and 14:00, and leaf samples (4–6 fascicles each) were weighed and placed either in 80% methanol in water (v/v) (for ABA analysis) or ethanol (for anatomical analysis). In addition, leaves were sampled for ABA analysis during the course of a previously conducted drought experiment (Gattmann et al., 2021). These leaf samples (*n* = 18 per treatment), initially snap-frozen in liquid nitrogen and stored at −80°C, were then transferred to 80% methanol in water (v/v) while still frozen for ABA analysis. During this previous drought experiment, midday leaf water potential (Ψ_leaf_) was intensively measured during the dry-down.

### Quantification of foliage ABA levels

Foliage ABA levels were quantified by physicochemical methods with an added internal standard. Samples were homogenized and 15 ng of [^2^H_6_]ABA was added to each sample as an internal standard. Endogenous ABA was extracted from homogenized tissue overnight at 4°C. An aliquot was taken and dried under vacuum until completeness. Samples were resuspended in 200 μL of 2% acetic acid in water (v/v), and hormone levels were quantified using liquid chromatography–mass spectrometry (Agilent 6400 series triple quadrupole LC/MS, USA) (McAdam and Brodribb, 2015).

### Epidermal and leaf cross-sectional anatomy

To assess changes in stomatal development, the cuticle morphology of the sampled leaves was studied. Briefly, leaf cuticles of the central 1 cm of a needle were prepared by making a longitudinal section through one corner of the leaf and then macerating the sample in aqueous chromium trioxide. Cuticles were mounted in glycerine jelly and imaged under 10× magnification for epidermal and stomatal cell density determination with care taken to avoid leaf margins, and 40× magnification for stomatal size measurements (AxiolmagerA2, Zeiss, Germany). Stomatal size was determined as the length of the stomatal complex. The mean stomatal and epidermal cell density of the whole leaf was quantified as the number of cells per square millimeter from five images per cuticle. SI, that is, the ratio of SD to ED was calculated for each image as follows:

(3) $$\mathrm{SI}\;(\%)=100\cdot \frac{\mathrm{SD}}{\mathrm{SD}+\mathrm{ED}}$$

Leaf width (LW) was measured on the same leaves using high-precision calipers (±0.01 mm). These leaves were also cross-sectioned to measure the shortest distance between the vein-to-the-stomata-bearing epidermis using a freezing stage microtome, stained with dilute aqueous toluene blue, and mounted in glycerine jelly for imaging as above.

### Hydraulic conductivity, xylem vulnerability curves, and wood anatomy

Branches for hydraulic analysis were taken in August and October 2019 from seedlings of the same population but were not part of the gas exchange measurements. These branch samples were immediately wrapped in foil and kept moist until analysis was conducted 2–3 days later. Maximal hydraulic conductivity (*K*_h_, kg m MPa^−1^ s^−1^) was measured in six stem segments (mean diameter *±* se: 7.33 ± 0.18 mm) per treatment after vacuum infiltration for 24 h at 30 mbar in the degassed solution of 10-mM KCl and 1-mM CaCl_2_ in demineralized water filtered to a particle size of 0.2 µm. The segments were recut underwater to a length of 73.91 ± 1.31 mm (mean ± se) with a sharp razor blade, connected to a Xyl’em Plus device (Bronkhorst France, Montigny les Cormeilles, France), and hydraulic conductivity was measured in the measurement solution described above. *K*_h_ was recorded at a pressure head of 4 kPa with the XylWin version 3.0 software (Bronkhorst France, Montigny les Cormeilles, France). Subsequently, we estimated specific conductivity (*K*_s_, kg m^−1^ MPa^−1^ s^−1^) from *K*_h_ divided by the cross-sectional area, and leaf-specific conductivity (*K*_l_, kg m^−1^ MPa^−1^ s^−1^) from *K*_h_ divided by the leaf area supported by the corresponding branch. The needle area of each branch was estimated from needle dry weight and treatment-averaged specific leaf area.

Xylem vulnerability curves were constructed for stem segments (aCO_2_: *n* = 5; eCO_2_: *n* = 6) with the flow-centrifuge technique (Cavitron; Cochard et al., 2005). Stem segments (mean basipetal diameter ± se: 7.25 ± 0.33 mm) were shorted to 27.5 cm under water and inserted in a custom-made rotor attached to an ultra-centrifuge (Sorvall RC-5C, Thermo Fisher Scientific, Waltham, MA, USA). Conductivity measurements started at −0.84 MPa and were repeated under increasingly negative water potentials until PLC (%) reached at least 90%. We fitted Weibull functions to describe the relationship between PLC and xylem pressure (Nadal-Sala et al., 2021).

From all segments used for xylem hydraulic measurements (aCO_2_: *n* = 12; eCO_2_: *n* = 13), semi-thin transverse sections were cut with a sliding microtome (G.S.L.1, Schenkung Dapples, Zurich, Switzerland), stained with safranin, and the complete cross-section digitalized at 100× magnification using a light microscope (Observer Z1, Carl Zeiss MicroImaging GmbH, Jena, Germany) equipped with an automated stage. Per segment, 51,242 ± 5,978 conduits (mean ± se) were analyzed on average. Image processing was performed with the open-source software Gimp (https://www.gimp.org) and ImageJ (Schneider et al., 2012) using the particle analysis function. Measured parameters included the conduit lumen-to-sapwood area ratio (*A*_lumen_, %), CD (n mm^−2^), and conduit diameter (*D*, μm) from major (1) and minor (2) conduit radii according to *D* =  ((32 ×  (a × b)^3^)/(a^2^ + b^2^))^¼^, the hydraulically weighted diameter (*D*_h_, μm) as *D*h = Σ*D*^5^/Σ*D*^4^ (Sperry and Saliendra, 1994) and potential hydraulic conductivity (*K*_p_, kg m^−1^ MPa^−1^ s^−1^) were calculated with the Hagen–Poiseuille equation as KP =  (*π* × *ρ* × Σ*D*^4^)/(128 *η* × *A*_xylem_), where *η* is the viscosity (1.002 10^−9^ MPa s) and *ρ* the density of water (998.2 kg m^−3^), both at 20°C, and *A*_xylem_ (m^2^) the sapwood area.

### Statistical analyses

Statistical analyses were conducted using R version 4.04 (R Core Team 2021). Differences in hydraulic parameters (*K*_s_, *K*_l_, and *A*_l_:*A*_s_), needle, and stomatal morphology were tested using nonparametric Mann–Whitney U tests. Wood anatomy parameters were assessed via GLS models (package nlme, Pinheiro et al., 2021). As wood anatomical traits closely covaried with branch thickness, their centered, natural log-transformed cross-sectional area was included as a covariate. Further, the residual variance was allowed to differ between treatments to account for inhomogeneous variances. We tested differences in the response of *g*_c_ to step-wise changes in CO_2_ concentrations (900, 400, or 200 ppm) using linear mixed effect models (package lme4; Bates et al., 2015) with tree as a random factor. The most parsimonious model was selected based on the Akaike information criterion.

We applied Bayesian statistics to address treatment differences of nonlinear relationships (BayesianTools package, Hartig et al., 2019). This included hydraulic vulnerability curves, responses of *g*_c_ with VPD, and Ψ_leaf_ and of ABA with Ψ_leaf_ (for model details see Supplemental Methods S1). We started with broad uniform but biologically meaningful priors (Supplemental Table S1) assuming a Gaussian likelihood and used a Differential-Evolution Markov Monte Carlo Chain with memory and a snooker update following the approach by Braak and Vrugt (2008). The posterior was obtained for each calibration (30,000 iterations) with a burn-in of 10,000 samples. We assessed between-chain convergence via the Gelman–Rubin diagnostic at ≤1.1 (Gelman and Rubin, 1992). In the case where we derived individual posteriors per tree seedling, these were later merged into a combined posterior distribution per treatment. Posterior predictive uncertainty was addressed by sampling 5,000 times the combined posterior. For each parameter, we report the median and 95% credible intervals. We considered differences between treatments to be meaningful if the CI between treatments did not overlap (see Supplemental Table S2 for model coefficients).

## Supplemental data

The following materials are available in the online version of this article.

**Supplemental Methods S1.** Nonlinear model fitting.

**Supplemental Table S1.** Prior distributions of the Bayesian model calibrations.

**Supplemental Table S2.** Parameter estimates of the Bayesian models.

**Supplemental Figure S1.** Leaf-level gas exchange.

**Supplemental Figure S2.** Midday leaf water potential during soil drought.

**Supplemental Figure S3.** Tree-level transpiration and photosynthesis.

## Supplementary Material

kiac482_Supplementary_Data

## Notes

M.G., D.Y., and N.K.R. designed the study. M.G., N.K.R., and B.B. conducted the experiments. R.L., D.N.S., S.A.M.M., and B.S. analyzed samples and contributed to data analyses. M.G. and N.K.R. wrote the manuscript with contributions from all co-authors.

The author responsible for distribution of materials integral to the findings presented in this article in accordance with the policy described in the Instructions for Authors (https://academic.oup.com/plphys/pages/general-instructions) is Nadine Ruehr (nadine.ruehr@kit.edu).
