# Supplementary material for: Anatomical adjustments of the tree hydraulic pathway decrease canopy conductance under long-term elevated CO2
Source: Plant Physiol. 2022 Oct 17;191(1):252–64. doi: 10.1093/plphys/kiac482 (PMC9806622; doi:10.1093/plphys/kiac482)
Supplement: kiac482_Supplementary_Data [file kiac482_supplementary_data.pdf]

Supplemental material to Gattmann et al. *Plant Physiology*

Supplemental Methods S1. Non-linear model fitting.

The following models were fit via Bayesian calibration as described in the main text. The number of accounted auto-correlation structures (number of seedlings measured) per model is given.

1) g_c response to increasing vapor pressure deficit (VPD)

For both treatments (aCO_2, $n = 6$ and eCO_2 $n = 6$) we assumed g_c to decline with VPD according to the following equation:

$$g_c = a_1 \mathrm{VPD}^{b_1} \quad \text{(S1)}$$

in which g_c is the canopy conductance in mol m^{-2} s^{-1}, VPD is the vapor pressure deficit in kPa, and a_1 and b_1 are the calibrated coefficients of the regression.

2) g_c response to declining midday leaf water potential (Ψ_{leaf})

For the two treatments (aCO_2 and eCO_2) we assumed a logistic decline of g_c following Ψ_{leaf} reductions, with a non-zero asymptote to represent minimum canopy conductance:

$$100 \cdot \left(\frac{g_c}{g_{c,\max}}\right) = a_2 + \frac{(100-a_2)}{1+\left(\frac{\Psi_{\mathrm{leaf}}}{b_2}\right)^{c_2}} \quad \text{(S2)}$$

in which $100 \cdot \left(\frac{g_c}{g_{c,\max}}\right)$ is the percentage of g_c with respect to the maximum canopy conductance, and Ψ_{leaf} is the midday leaf water potential (MPa). Regarding the calibrated coefficients, a_2 is the percent of stomatal conductance relative to the maximum at the asymptote, which is the equivalent of minimum g_c. b_2 is the leaf water potential at which the percent of g_c relative to maximum g_c is $\frac{100+a_2}{2}$, and c_2 is a scaling factor.

3) ABA response to declining midday leaf water potential (Ψ_{leaf})

For the two treatments (aCO_2 and eCO_2) we assumed a potential increase of ABA concentration with declining Ψ_{leaf} as follows:

$$\mathrm{ABA} = a_4 \cdot (-\Psi_{\mathrm{leaf}})^{b_4} \quad \text{(S4)}$$

in which ABA is the concentration of abscisic acid in the leaves (ng g^{-1}), Ψ_{leaf} is the midday leaf water potential (MPa), and a_4 and b_4 are the calibrated coefficients for the regression.

4) Percent loss in conductance with declining xylem water potential (Ψ_{xylem})

For both treatments (aCO_2 $n = 5$ and eCO_2 $n = 6$) we fitted a cumulative probability function in form of a Weibull distribution:

$$PLC = 100 \cdot \left(1 - \exp\left(-\left(\frac{\Psi_{\mathrm{xylem}}}{a_5}\right)^{b_5}\right)\right) \quad \text{(S5)}$$

with PLC is the percent loss of hydraulic conductance (%),Ψ_{xylem} is the xylem water potential (MPa), a_5 and b_5 are the calibrated coefficients, where a_5 is a scale parameter of reference xylem water potential value (MPa), and b_5 a shape parameter.

Supplemental Table S1. Prior distributions of the Bayesian model calibrations. All priors are assumed to follow a uniform distribution, with minimum (min) and maximum (max) values as reported in the respective columns.

Model	Parameter	Distribution	Min	Max
ABA vs Ψ_{leaf}	*a*	Uniform	50	500
	b	Uniform	0.1	2.5
PLC vs Ψ_{xylem}	*a*	Uniform	-6.5	-2.5
	b	Uniform	1	8
g_c vs VPD	*a*	Uniform	-3.5	0.5
	b	Uniform	-4	-0.1
$g_{c\ rel}$ vs Ψ_{leaf}	*a*	Uniform	1	5
	b	Uniform	-0.5	-2
	c	Uniform	1.5	6

Supplemental Table S2. Parameter estimates of the Bayesian models. Model coefficients are given per treatment (eCO_2 = elevated CO_2, aCO_2 = ambient CO_2) and during decreasing CO_2 (from 900 to 400 to 200 ppm) in the eCO_2 seedlings. All parameter values are reported as the median and the 95% credible intervals per treatment. Bold letters indicate non-overlapping credible intervals between treatments for a given test. ABA is abscisic acid concentration (ng g^{-1}), Ψ_{leaf} is midday leaf water potential (MPa), PLC is percent loss in xylem hydraulic conductance (%). Ψ_{xylem} is xylem water potential (MPa), g_c is canopy conductance (mol m^{-2} s^{-1}), PAR is photosynthetic active radiation (μmol m^{-2} s^{-1}), VPD is vapor pressure deficit (kPa) and $g_{c,rel}$ is canopy conductance relative to the treatment-specific maximum canopy conductance (%).

		2.5%CI	Median	97.5%CI	2.5%CI	Median	97.5%CI	2.5%CI	Median	97.5%CI
	Treatment		Coef *a*			Coef *b*				
ABA vs Ψ_{leaf}	eCO_2	62	159.6	283.4	0.99	1.37	1.93			
	aCO_2	181.3	248.9	321.9	0.85	1.02	1.21			
			Coef *a*			Coef *b*				
PLC vs Ψ_{xylem}	eCO_2	-6.48	-5.35	-4.55	2.67	4.26	5.29			
	aCO_2	-6.47	-5.38	-4.58	3.57	4.66	7.99			
			Coef *a*			Coef *b*				
g_c vs VPD	eCO_2	**0.065**	**0.087**	**0.109**	-2.63	-2.33	-2.05			
	aCO_2	**0.173**	**0.202**	**0.405**	-2.48	-1.68	-1.37			
			Coef *a*			Coef *b*			Coef *c*	
g_{c-rel} vs Ψ_{leaf}	eCO_2	2.29	2.93	3.57	-1.22	-1.2	-1.7	4.11	4.47	4.89
	aCO_2	2.11	2.76	3.39	-1.27	-1.24	-1.21	4.31	4.64	5.11

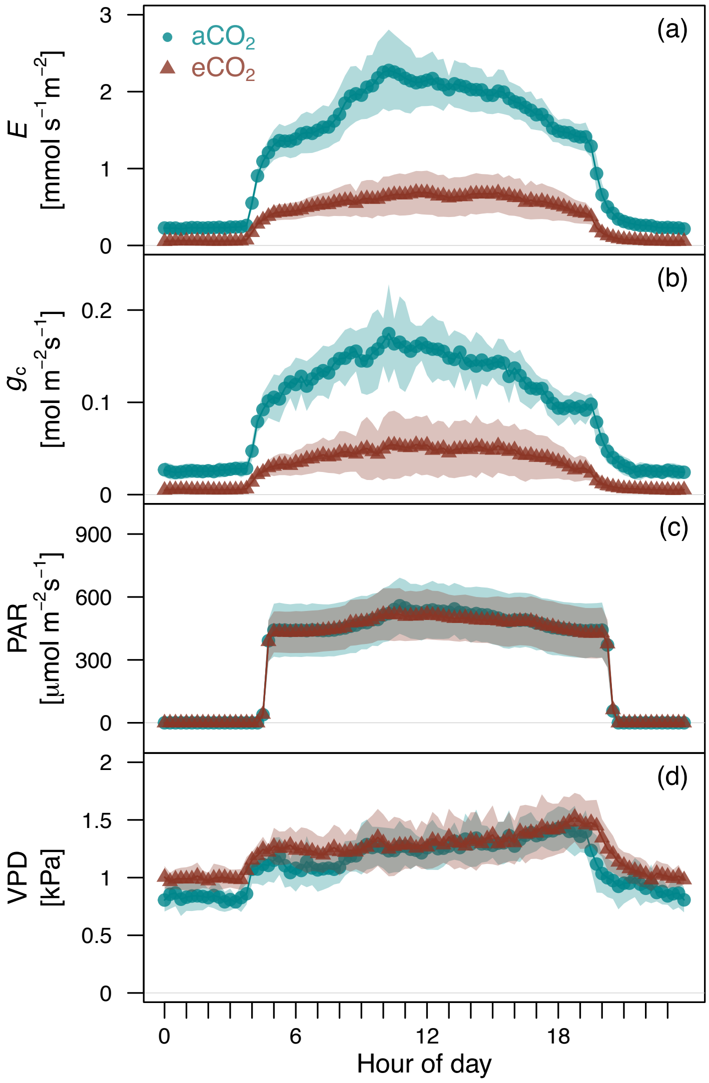

Supplemental Figure S1. Leaf-level gas exchange. Diurnal course of leaf-level transpiration (*E*) (a) and canopy conductance (g_c) (b) as well as photosynthetic active radiation (PAR) (c) and vapor pressure deficit (VPD) (d) under ambient (aCO_2) and elevated [CO_2] (eCO_2). Shown are quarter-hourly treatment means over three (aCO_2) and four (eCO_2) days of acclimation with shaded areas depicting ± standard deviation (*n* = 6 seedling per treatment).

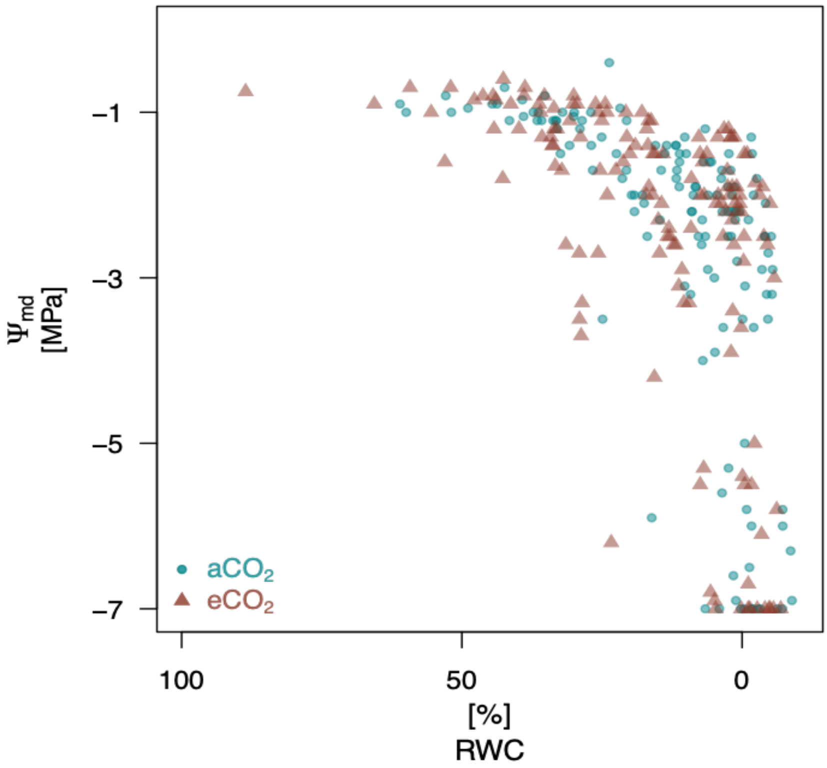

Supplemental Figure S2. Midday leaf water potential during soil drought. Midday leaf water potential (Ψ_{md}) versus daily-averaged relative soil water content (RWC) during the course of a lethal soil drought for aCO2 and eCO2 Aleppo pine seedlings (*n* = 18 per treatment). Shown are individual Ψ_{md} measurements (aCO_2: solid points; eCO_2: solid triangles). See also Gattmann et al. (2021).

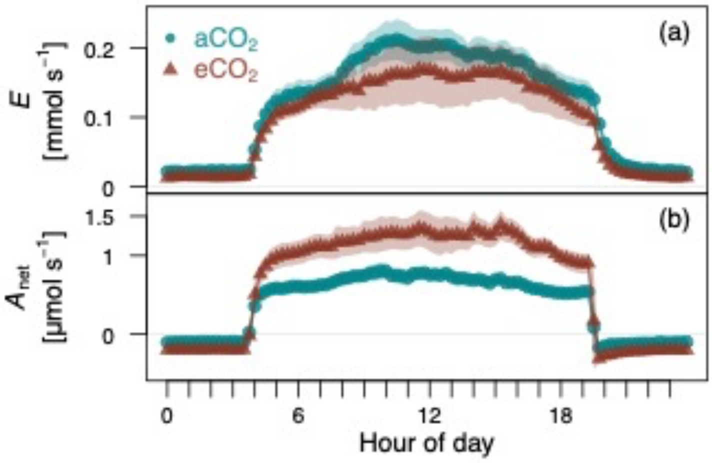

Supplemental Figure S3. Tree-level transpiration and photosynthesis**.** Diurnal course of tree-level transpiration (*E*) (a) and tree-level net photosynthesis (A_{net}) under ambient (aCO_2) and elevated CO_2 (eCO_2). Shown are quarter-hourly treatment means over three (aCO_2) and four (eCO_2) days of acclimation with shaded areas depicting ± standard deviation (*n* = 6 seedlings per treatment).
